# Altered Default Mode Network Is Associated With Cognitive Impairment in CADASIL as Revealed by Multimodal Neu**r**oimaging

**DOI:** 10.3389/fneur.2021.735033

**Published:** 2021-12-06

**Authors:** Panlong Li, Qi Huang, Shiyu Ban, Yuan Qiao, Jing Wu, Yu Zhai, Xiaoxia Du, Fengchun Hua, Jingjing Su

**Affiliations:** ^1^School of Electrical and Information Engineering, Zhengzhou University of Light Industry, Zhengzhou, China; ^2^Positron Emission Tomography (PET) Center, Huashan Hospital, Fudan University, Shanghai, China; ^3^Shanghai Key Laboratory of Magnetic Resonance and Department of Physics, School of Physics and Materials Science, East China Normal University, Shanghai, China; ^4^Department of Neurology, Shanghai Ninth People's Hospital, Shanghai Jiao Tong University School of Medicine, Shanghai, China; ^5^Department of Nuclear Medicine, Longhua Hospital, Shanghai University of Traditional Chinese Medicine, Shanghai, China

**Keywords:** CADASIL, default mode network, cognitive impairment, MRI, PET

## Abstract

**Background and Purpose:** Cerebral autosomal dominant arteriopathy with subcortical infarcts and leukoencephalopathy caused by mutations in the NOTCH3 gene is a hereditary cerebral small vessel disease, manifesting with stroke, cognitive impairment, and mood disturbances. Functional or structural changes in the default mode network (DMN), which plays important role in cognitive and mental maintenance, have been found in several neurological and mental diseases. However, it remains unclear whether DMN is altered in patients with cerebral autosomal dominant arteriopathy with subcortical infarcts and leukoencephalopathy (CADASIL).

**Methods:** Multimodal imaging methods, including MRI and positron emission tomography (PET), were applied to evaluate the functional, structural, and metabolic characteristics of DMN in 25 patients with CADASIL and 42 healthy controls.

**Results:** Compared with controls, patients with CADASIL had decreased nodal efficiency and degree centrality of the dorsal medial pre-frontal cortex and hippocampal formation within DMN. Structural MRI and diffusion tensor imaging (DTI) showed decreased gray matter volume and fiber tracks presented in the bilateral hippocampal formation. Meanwhile, PET imaging showed decreased metabolism within the whole DMN in CADASIL. Furthermore, correlation analyses showed that these nodal characteristics, gray matter volume, and metabolic signals of DMN were related to cognitive scores in CADASIL.

**Conclusions:** Our results suggested that altered network characteristics of DMN might play important roles in cognitive deficits of CADASIL.

## Introduction

Cerebral autosomal dominant arteriopathy with subcortical infarcts and leukoencephalopathy is the most common hereditary cerebrovascular disease (1). It is caused by the pathogenic mutations in the NOTCH3 gene on chromosome 19 and, therefore, shows familial inheritance. The typical clinical manifestations of cerebral autosomal dominant arteriopathy with subcortical infarcts and leukoencephalopathy (CADASIL) are migraine with aura, stroke, mood disturbances, and progressive cognitive impairments, including deficits in executive function, processing speed, attention, and memory (2).

Default mode network is a notable network that shows greater activity during the resting state than when performing tasks. It was first addressed by Raichle in a positron emission tomography (PET) study in 2001 (3). The component brain areas of default mode network (DMN) mainly include posterior cingulate cortex and precuneus (PCC/PCU), medial pre-frontal cortex (MPFC), medial and inferior temporal lobes, and inferior parietal lobe (IPL), and play important roles in a great variety of cognitive domains, such as working memory, visuomotor, visual language, and mental imagery (4). DMN has become a central research theme in neuropsychiatric disorders, including stroke, dementia, migraine, traumatic brain injury, depression, anxiety, and schizophrenia (5–10). A series of important research results about changed DMN characteristics in these disorders have been reported by using magnetic resonance imaging (MRI) and PET methods (5–10). However, as a focus of research into cognition, whether DMN is altered in patients with CADASIL remains unclear.

Several studies have demonstrated brain alterations in functional and structural imaging parameters in patients with CADASIL. Our previous resting-state functional MRI (fMRI) studies showed that altered functional activity and connectivity in PCC/PCU and para-hippocampal cortex (PHC) were associated with cognitive impairment in CADASIL (11, 12). Diffusion tensor imaging (DTI) studies have demonstrated widespread white matter lesions associated with cognitive deficits in CADASIL (13–15). Moreover, a case study using diffusion tensor tractography indicated that neural tract injuries were mainly located in the frontal lobe in a patient with CADASIL (16). Furthermore, a recent ^18^F-2-fluoro-2-deoxy-d-glucose PET (^18^F-FDG PET) study showed that decreased metabolism in the limbic lobe, including the hippocampus and PHC, was positively associated with a cognitive score in patients with CADASIL (17). Several regions involved in CADASIL belong to the hub nodes of DMN, and the DMN has a high degree of connectivity across the above-involved regions, including the PCC/PCU, MPFC, hippocampus, and PHC (5, 18). Though promising initial results from different neuroimaging studies have shown considerable overlap with areas typically considered part of the DMN, few studies explored the DMN in patients with CADASIL. Thus, based on the brain regions highlighted in these CADASIL studies, we assumed DMN modifications in patients with CADASIL.

Therefore, in the present study, we focused on multimodal imaging outcomes of DMN in CADASIL. To characterize the DMN comprehensively, resting-state fMRI, T1-weighted MRI, DTI, and ^18^F-FDG PET were employed to assess functional network properties, gray matter volume (GMV), structural connectivity, and metabolism in DMN, and the associations with cognitive deficits.

## Materials and Methods

### Participants

The study protocol was approved by the ethics committee in the Shanghai Ninth People's Hospital. All participants were fully informed of the study procedures and signed the informed consent. A total of 25 patients with CADASIL from 14 families evaluated at Shanghai Ninth People's Hospital between May 2016 and January 2019 were recruited for this study. For all patients, the diagnosis was confirmed by the identification of pathogenic mutations in the NOTCH3 gene (19). All subjects underwent detailed standard neurological examinations. Subjects were excluded from the study if they had severe depression or anxiety according to evaluation by two trained neuropsychologists using the Hamilton Depression Scale (HAMD) and the Hamilton Anxiety Scale (HAMA) (20, 21). Subjects were diagnosed with severe depression and anxiety based on HAMD and HAMA scores >17 and >14, respectively. Neurological deficits in all subjects were assessed using the National Institutes of Health Stroke Scale (NIHSS) and the modified Rankin scale (mRs). Cognitive scores in all subjects were recorded by the Montreal Cognitive Assessment (MoCA) and Mini-Mental State Examination (MMSE). Most of the patients underwent both MRI and PET/computed tomography (PET/CT), but four underwent only MRI and another four underwent only PET/CT.

Forty-two healthy subjects were recruited as a control group based on the following criteria: no history of stroke, headache, cognitive impairment, or vascular disease risk factors; no family history of cerebrovascular diseases or vascular disease risk factors; not taking medications and no substance addiction, such as drugs, cigarettes, or alcohol. All of the healthy subjects had normal results on neurological and general examinations. Among the 42 controls, 21 underwent only MRI and the remaining 21 controls underwent only PET/CT. The sample size and demographic information of each group were listed in Table 1.

**Table 1:** Demographic information of CADASIL and control.

	**CADASIL (*****n*** **= 25)**		**Controls (*****n*** **= 42)**		***p*** **-values**	
	**MRI (*****n*** **= 21), PET (*****n*** **= 21)**		**MRI (*****n*** **= 21), PET (*****n*** **= 21)**		***p*1**	***p*2**
Male/female	13/8	14/7	13/8	14/7	1	1
Age (years), mean ± SD	48.4 ± 14.2	46.3 ± 14.0	48.7 ± 14.3	45.8 ± 12.1	0.9	0.9
Education (years), mean ± SD	8.7 ± 3.5	8.9 ± 3.3	9.1 ± 3.2	9.3 ± 3.0	0.8	0.8
Family history, *n* (%)	20 (95.2)	21 (100)	–	–	–	–
Migraine, *n* (%)	3 (14.3)	3 (14.3)	–	–	–	–
Migraine with aura, *n* (%)	2 (9.5)	2 (9.5)	–	–	–	–
Migraine without aura, *n* (%)	1 (4.8)	1 (4.8)	–	–	–	–
WMH volume (cm^3^), mean ± SD (*n* = 19)	78.3 ± 55.7	–	–	–	–	–
Lacunar volume (cm^3^), mean ± SD (*n* = 17)	1.9 ± 1.8	–	–	–	–	–
Microbleeds (number), mean ± SD (*n* = 6)	6.0 ± 1.8	–	–	–	–	–
O'Sullivan sign, *n* (%)	9 (42.9)					
HAMD, median ± IQR	4 ± 6	4 ± 5	3 ± 4	3 ± 3	0.3	0.3
HAMA, median ± IQR	3 ± 1	3 ± 1	3 ± 1	3 ± 2	0.7	0.7
NIHSS, median ± IQR	0 ± 1	0 ± 1	0 ± 0	0 ± 0	0.01	0.006
mRs, median ± IQR	1 ± 2	1 ± 2	0 ± 0	0 ± 0	0.001	0.001
MoCA, median ± IQR	21 ± 15	20 ± 11	26 ± 3	27 ± 1	0.000	0.000
MMSE, median ± IQR	24 ± 12	24 ± 11	28 ± 1	28 ± 2	0.001	0.002

*SD and IQR represent standard deviation and interquartile range, respectively. P1 represents p-values of the comparisons between patients and controls in the MRI group, and p2 represents p-values of the comparisons between patients and controls in the PET group. CADASIL, cerebral autosomal dominant arteriopathy with subcortical infarcts and leukoencephalopathy; MRI, magnetic resonance imaging; PET, positron emission tomography; WMH, white matter hyperintensities; TIA, transient ischemic attack; HAMD, Hamilton Depression Scale; HAMA, Hamilton Anxiety Scale; NIHSS, National Institute of Health Stroke Scale; mRS, modified Rankin scale; MoCA, Montreal Cognitive Assessment; MMSE, Mini-Mental State Examination*.

### MRI Acquisition

Subjects in the first control group and 21 patients with CADASIL underwent MRI, including resting-state fMRI, structural MRI (T1-weighted, T2-weighted, and fluid-attenuated inversion recovery [FLAIR] imaging), and DTI on a 3 Tesla system (Trio Tim; Siemens Healthcare, Malvern, PA, USA) with a 12-channel head coil at East China Normal University. Soft earplugs and custom-fit foam were applied to reduce noise and movement artifacts. Resting-state fMRI was performed using a T2^*^-weighted gradient-echo echo-planar imaging pulse sequence with the following parameters: repetition time/echo time (TR/TE) = 2,000/30 ms; flip angle = 90°; field of view (FOV) = 220 mm × 220 mm; number of slices = 33; resolution = 3.44 mm × 3.44 mm × 4.38 mm; total volume = 210. The fMRI acquisition time was 7 min and 6 s for each subject. During the fMRI scan, the subjects kept their eyes closed but did not fall asleep. The whole-brain anatomical volume was obtained using a high-resolution T1-weighted 3D magnetization-prepared rapid-acquisition gradient-echo pulse sequence with the following parameters: TR = 2,530 ms; TE = 2.34 ms; flip angle = 7°; FOV = 256 mm × 256 mm; number of slices = 192; resolution = 1 mm × 1 mm × 1 mm. The T1-weighted image acquisition time was 6 min and 3 s for each subject. T2-weighted imaging was obtained using turbo spin-echo dark fluid sequence with the following parameters: TR/TE = 5,500/83 ms; FOV = 220 mm × 220 mm; number of slices = 35; resolution = 0.38 mm × 0.38 mm × 5.2 mm. The T2-weighted image scanning time was 1 min and 26 s for each subject. The parameters of FLAIR imaging were: TR/TE = 9,000/93 ms; FOV = 220 mm × 220 mm; number of slices = 30; resolution = 0.43 mm × 0.43 mm × 4.55 mm. The FLAIR image scanning time was 4 min and 50 s for each subject. DTI was performed using a single-shot, spin-echo planar imaging sequence acquired in contiguous axial planes with the following parameters: 64 non-collinear directions, diffusion weighting of b = 1,000 s/m^2^, an acquisition without diffusion weighting of b = 0; TR/TE = 8,900/86 ms; FOV = 256 mm × 256 mm, covered the whole brain; 70 contiguous slices; resolution = 2 mm × 2 mm × 2 mm. The DTI scanning time was 10 min and 7 s for each subject. The total MRI acquisition time was 29 min and 38 s for each subject.

### PET Acquisition

^18^F-2-fluoro-2-deoxy-d-glucose PET data were acquired using a Siemens Biograph Truepoint HD 64 PET/CT is made by Simens in Germany at the PET Center of Huashan Hospital, Fudan University. ^18^F-FDG was synthesized and radiolabeled at the PET Center according to the protocol of the manufacturer under the inspection by the Chinese Food and Drug Administration. Before ^18^F-FDG injection, subjects were asked to avoid strenuous physical activity and fast for about 6 h to maintain blood glucose level <8 mmol/L. After receiving an injection of ^18^F-FDG at a dose of 5.55 MBq/kg (0.15 mCi/kg), subjects rested in a dimly lit room for 50 min. Before PET acquisition, a low-dose CT scan was performed for attenuation correction, and then 10-min PET images were reconstructed using a filtered back-projection algorithm. The matrix size of the reconstructed images was 168 × 168 × 148 with a resolution of 2.04 mm × 2.04 mm × 1.5 mm.

### Data Processing

Prior to preprocessing, all the raw DICOM data were converted to the Neuroimaging Informatics Technology Initiative format (NII) using MRICRON software (https://people.cas.sc.edu/rorden/mricron/install.html) and the quality of the images was checked visually.

#### fMRI Data Processing

The resting-state fMRI data were preprocessed using Data Processing Assistant for Resting-State fMRI (DPARSF; http://www.restfmri.net) (22, 23). Data were preprocessed starting with the removal of the first 10 volumes to ameliorate possible effects of scanner instability and the adaptation of subjects to the environment. Then, slice time correction was applied to reduce the effects of within-scan acquisition time differences between slices. To correct the effects of head motion, the fMRI images of each subject were realigned and registered. All subjects had head motions <1.5° of rotation or 0.5 mm of mean frame-wise displacement (24). The fMRI images were then normalized into the Montreal Neurological Institute (MNI) space using the EPI template and smoothed by a full-width at half-maximum (FWHM) 8 mm Gaussian kernel. Following spatial smoothing, linear detrend was performed to remove noise due to long-term physiological shifts, movement-related noise remaining after realignment, and instrumental instability. To reduce further the effects of noise, the fMRI images were filtered with a temporal band-pass filter (0.01–0.08 Hz). Finally, the six head motion parameters, global mean signal, white matter signal, and cerebrospinal fluid signal were regressed out as nuisance covariates to remove these unwanted signals.

With reference to previous studies (18, 25, 26), 11 separate regions comprising the left DMN and 11 mirrored regions comprising the right DMN were defined as regions of interest (ROIs). The 11 ROIs were spheres of radius 8 mm in the dorsal MPFC (dMPFC), anterior MPFC (aMPFC), ventral MPFC (vMPFC), posterior IPL (pIPL), temporal-parietal junction (TPJ), lateral temporal cortex (LTC), temporal pole (TempP), PCC, retrosplenial cortex (RSC), PHC, and hippocampal formation (HF) (see Supplementary Table 1 and Supplementary Figure 1 for coordinates and spatial positions). Average fMRI time-series were calculated across every voxel in each ROI. The absolute value of Fisher's z-transformed Pearson's correlation coefficient between each pair of time-series was defined as the functional connectivity (FC) strength.

Graph analysis of the pairwise (11 × 11) correlation matrixes was performed using GRETNA (v2.0.0; https://www.nitrc.org/projects/gretna/) (27). Global and nodal network properties, including nodal degree centrality, nodal shortest path length, nodal clustering coefficient, nodal efficiency, nodal local efficiency, betweenness centrality, global efficiency, assortativity coefficient, and small-worldness, were calculated to delineate the integrative and local topological architecture of the DMN, respectively. Their definitions and calculations of the nodal and global network properties were summarized in Supplementary Table 2.

#### T1-Weighted Data Processing

The T1-weighted MRI data were preprocessed using the Computational Anatomy Toolbox (CAT12; http://dbm.neuro.uni-jena.de/cat12) implemented in statistical parametric mapping software (SPM12; htttp://www.fil.ion.ucl.ac.uk/spm/). First, all T1-weighted MRI data were normalized into the MNI space using the Diffeomorphic Anatomic Registration Through Exponentiated Lie algebra algorithm (DARTEL). The bias field inhomogeneities were corrected to remove non-uniform intensities. Normalized images were then segmented into gray matter, white matter, and cerebrospinal fluid components. The total intracranial volume (TIV) of each participant was evaluated to correct for the effects of differences in brain size. The internal gray matter threshold was set to 0.2 to exclude artifacts on the gray-white matter border. Thereafter, all preprocessed scans were smoothed with the FWHM 6 mm Gaussian kernel. Finally, the average GMV was calculated across every voxel in each ROI.

#### DTI Data Processing

The raw DTI data were preprocessed using FMRIB Software Library (FSL; http://www.fmrib.ox.ac.uk/fsl/index.html.) First, eddy current correction was performed to correct for head motion artifacts and eddy current distortions. Then, the brain of each subject was extracted using the FSL Brain Extraction Tool (BET). Tensor reconstruction and fiber tracking were applied by Diffusion Toolkit TrackVis (https://www.nitrc.org/projects/trackvis.) The Fiber Association Continuous Tracking (FACT) algorithm in Diffusion Toolkit was applied to obtain the whole-brain fiber tracts. The main parameters in fiber tractography were as follows: maximum turning angle threshold at 35°; minimum fractional anisotropy (FA) threshold of 0.2. Then, SPM12 was applied to bring all the individual tracts into the MNI space by non-linear transformation methods. In the normalization step, tracts were spatially normalized by: co-registering T1-weighted MRI to the corresponding FA image; calculating the deformation field of the individual coregistered T1-weighted image space to the MNI space; applying the deformation field to tracts and bringing them into the MNI space. Thereafter, TrackVis was used to record the number of tracts (NT) passing through each ROI.

#### PET Data Processing

First, PET images of each subject were processed using SPM12 software with spatial normalization and smoothing. The PET template in SPM12 was used in the spatial normalization step. The FWHM 8 mm Gaussian kernel was applied in the smoothing step. The average glucose metabolism was then calculated across every voxel in each ROI.

### Statistical Analysis

Statistical analysis was performed using IBM SPSS Statistics for Windows (SPSS, Chicago, IL, USA). The Chi-square tests and permutation tests (permutation times = 10,000) were used to compare demographic, clinical, and imaging characteristics between the CADASIL and control groups, as appropriate. Furthermore, the two-sample *t-*test was applied for voxel-wise metabolism comparisons between the CADASIL and corresponding control groups using SPM12 software. Subsequently, partial correlations were established to estimate the relations between the cognitive deficits and the imaging characteristics showing significant between-group differences. Age, sex, and education levels were entered as covariates in partial correlation analysis. Benjamini-Hochberg false discovery rate (FDR) correction was further used to avoid type-I errors in the multiple comparisons and correlations. The results of two-sample comparisons and partial correlations were regarded as significant at *p* < 0.05 (two-tailed) with FDR correction.

## Results

### Demographic and Clinical Data

Table 1 showed comparisons of the demographic and clinical data of the CADASIL and healthy control groups. There were significant differences between the two groups in terms of neurological deficits and cognitive scores, but no differences in sex, age, education levels, or depression and anxiety symptom scores.

### Network Analysis of DMN

**Figure 1:**
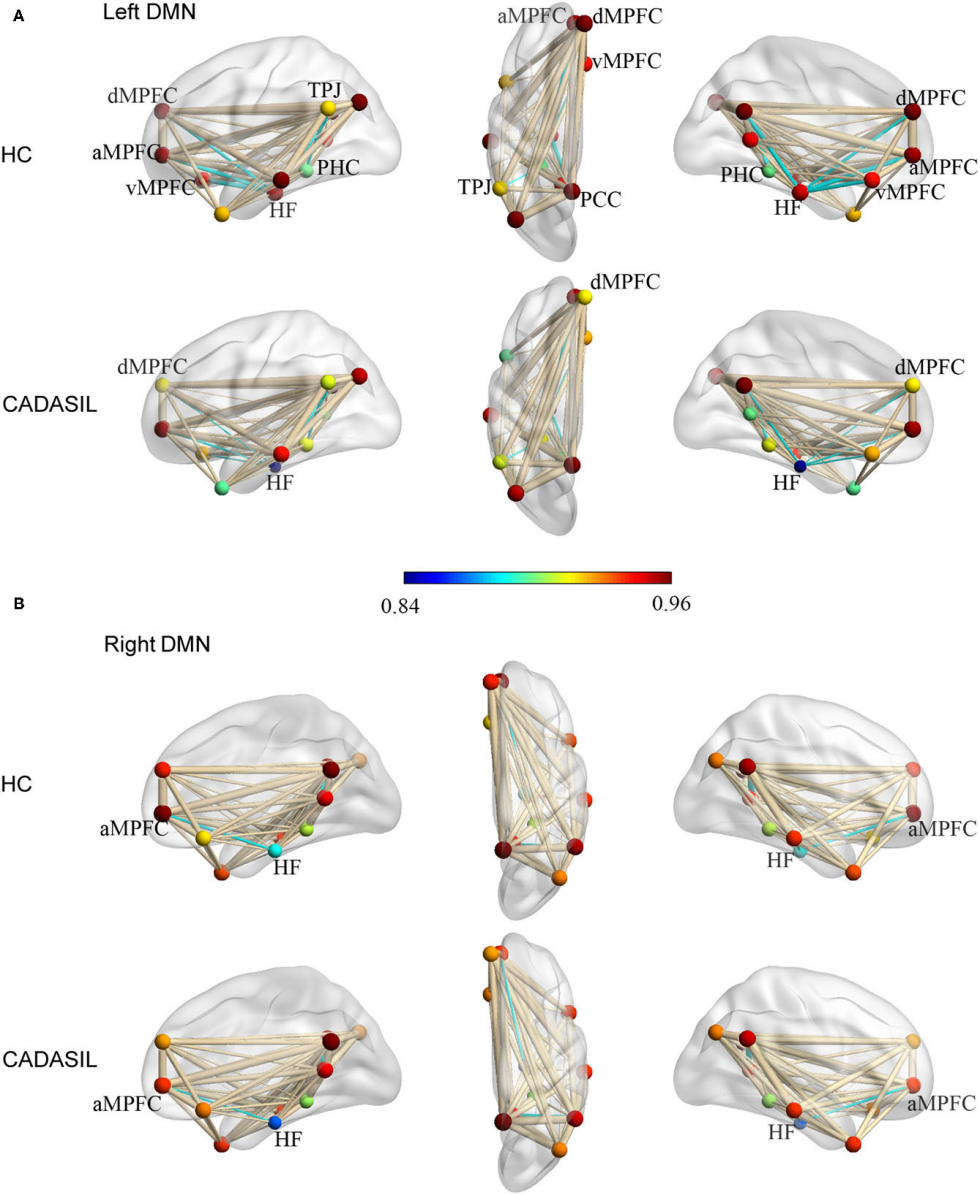
Topological properties of DMN in CADASIL and control groups in the left **(A)** and right **(B)** hemisphere. The color bar corresponded to nodal color, representing the mean nodal efficiency in the corresponding group. Nodal size represented the mean degree of centrality in the corresponding group. The thickness of edges reflected the strength of FC between regions. FC showing significant differences between the two groups was indicated in cyan (*p* < 0.05). DMN, default mode network; CADASIL, cerebral autosomal dominant arteriopathy with subcortical infarcts and leukoencephalopathy; FC, functional connectivity; HC, healthy control; a MPFC, anterior medial pre-frontal cortex; dMPFC, dorsal medial pre-frontal cortex; vMPFC, ventral medial pre-frontal cortex; HF, hippocampal formation; PCC, posterior cingulate cortex; PHC, parahippocampal cortex; TPJ, temporal-parietal junction.

**Figure 2:**
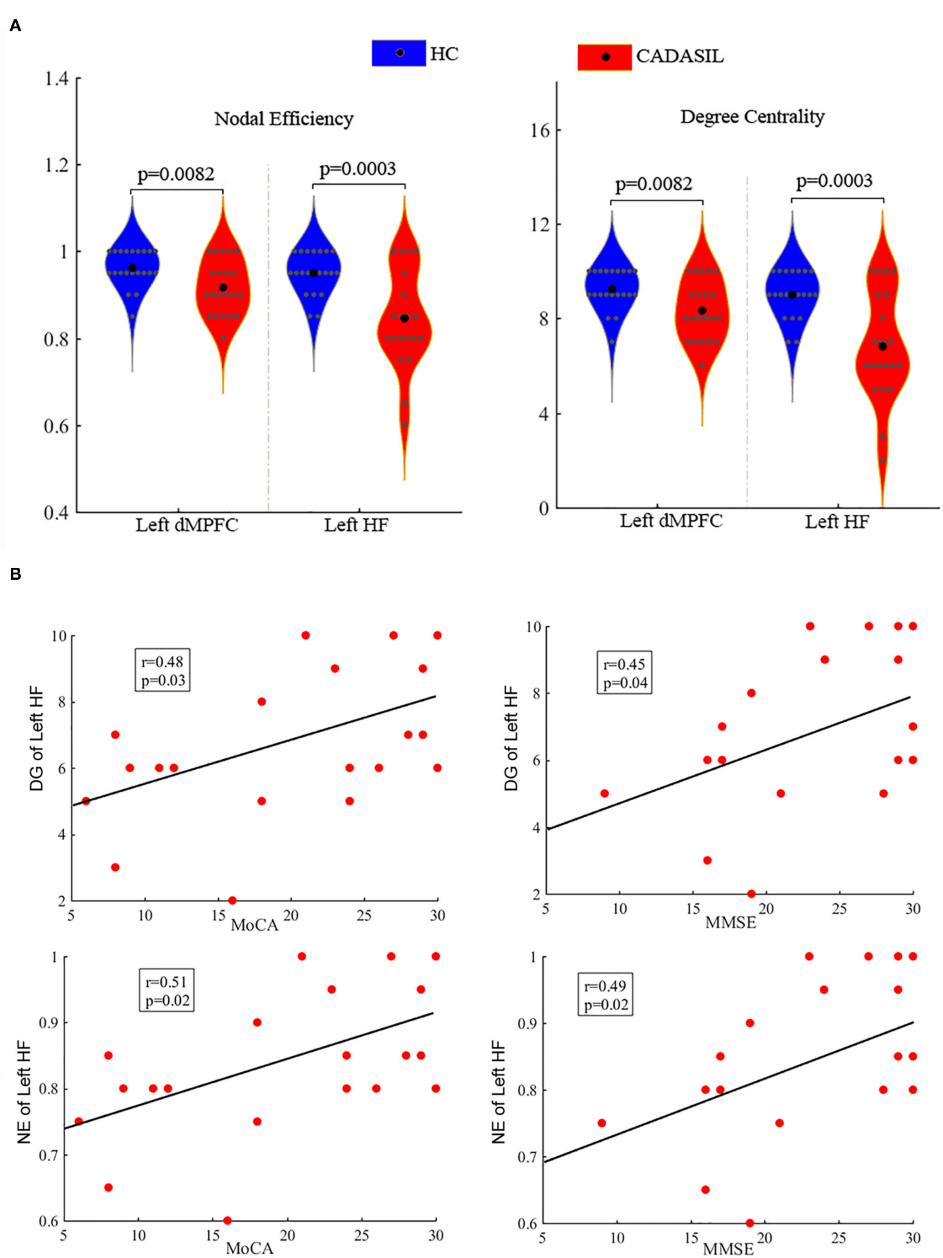
Differences between CADASIL and control groups in topological properties and correlations between the properties and cognitive scores in the DMN. **(A)** Nodal efficiency and degree centrality were significantly different between the CADASIL and healthy control groups in the left DMN. The CADASIL had decreased nodal efficiency and degree centrality in the left dMPFC and HF. **(B)** Linear correlations between network properties in the DMN and cognitive scores in CADASIL. CADASIL, cerebral autosomal dominant arteriopathy with subcortical infarcts and leukoencephalopathy; DMN, default mode network; dMPFC, dorsal medial pre-frontal cortex; HF, hippocampal formation; HC, healthy control; DG, degree centrality; NE, nodal efficiency; MoCA, Montreal Cognitive Assessment; MMSE, Mini-Mental State Examination.

Compared with the healthy control group, the CADASIL group had decreased FC between the HF and the MPFC (aMPFC, dMPFC, and vMPFC), as well as increased FC between the TPJ and PHC within the left DMN (Figure 1A and Supplementary Figure 2). Further network analysis showed that the nodal characteristics of the left dMPFC and HF, including nodal efficiency and degree centrality, were significantly different between the CADASIL and healthy control groups (Figure 2A). The integrative topological architecture of the left DMN was not significantly different between the two groups.

In the right DMN, the CADASIL group showed decreased FC between the HF and the aMPFC as well as the dMPFC, and increased FC between the TPJ and RSC in comparison to the healthy control group (Figure 1B and Supplementary Figure 2). There were no significant differences in the global or local topological architecture of the right DMN between the two groups.

### GMV Analysis of DMN

**Figure 3:**
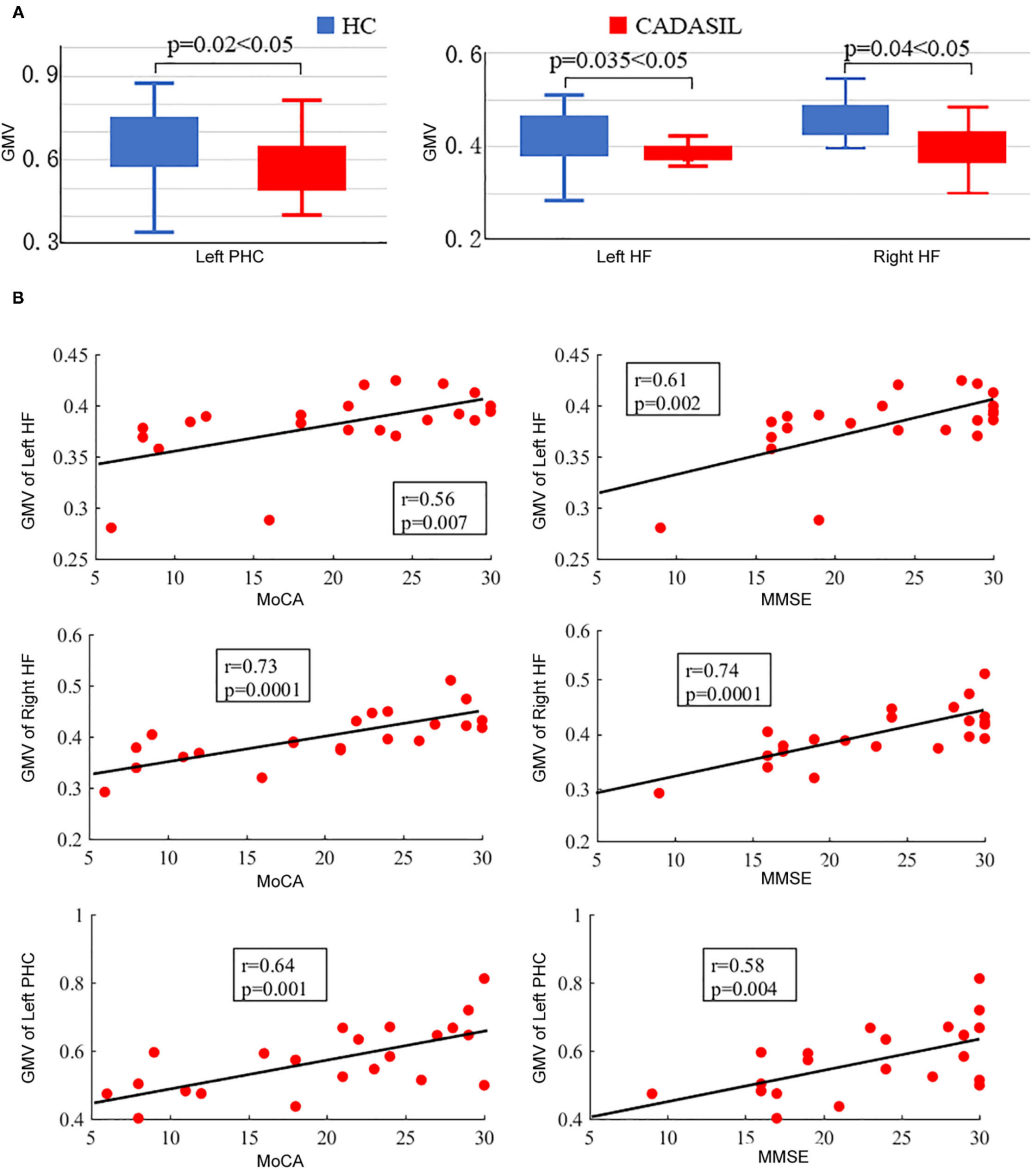
Results of GMV analysis in CADASIL and control groups. **(A)** Alterations in GMV of the DMN in CADASIL. Patients with CADASIL had decreased GMV in the left PHC and bilateral HF compared with healthy controls. **(B)** Correlations between GMV of ROIs within the DMN and cognitive scores in CADASIL. GMV, gray matter volume; CADASIL, cerebral autosomal dominant arteriopathy with subcortical infarcts and leukoencephalopathy; DMN, default mode network; PHC, parahippocampal cortex; HF, hippocampal formation; ROI, region of interest; HC, healthy control; MoCA, Montreal Cognitive Assessment; MMSE, Mini-Mental State Examination.

There was no significant difference in TIV between the CADASIL group and the healthy control group. Compared with the healthy control group, the patients with CADASIL had decreased GMV in the left PHC (0.58 ± 0.1 vs. 0.66 ± 0.12, respectively, *p* = 0.02) and bilateral HF (left: 0.38 ± 0.04 vs. 0.41 ± 0.05, respectively, *p* = 0.035; right: 0.40 ± 0.05 vs. 0.44 ± 0.06, respectively, *p* = 0.04) (Figure 3A).

### Fiber Tracks Analysis of DMN

**Figure 4:**
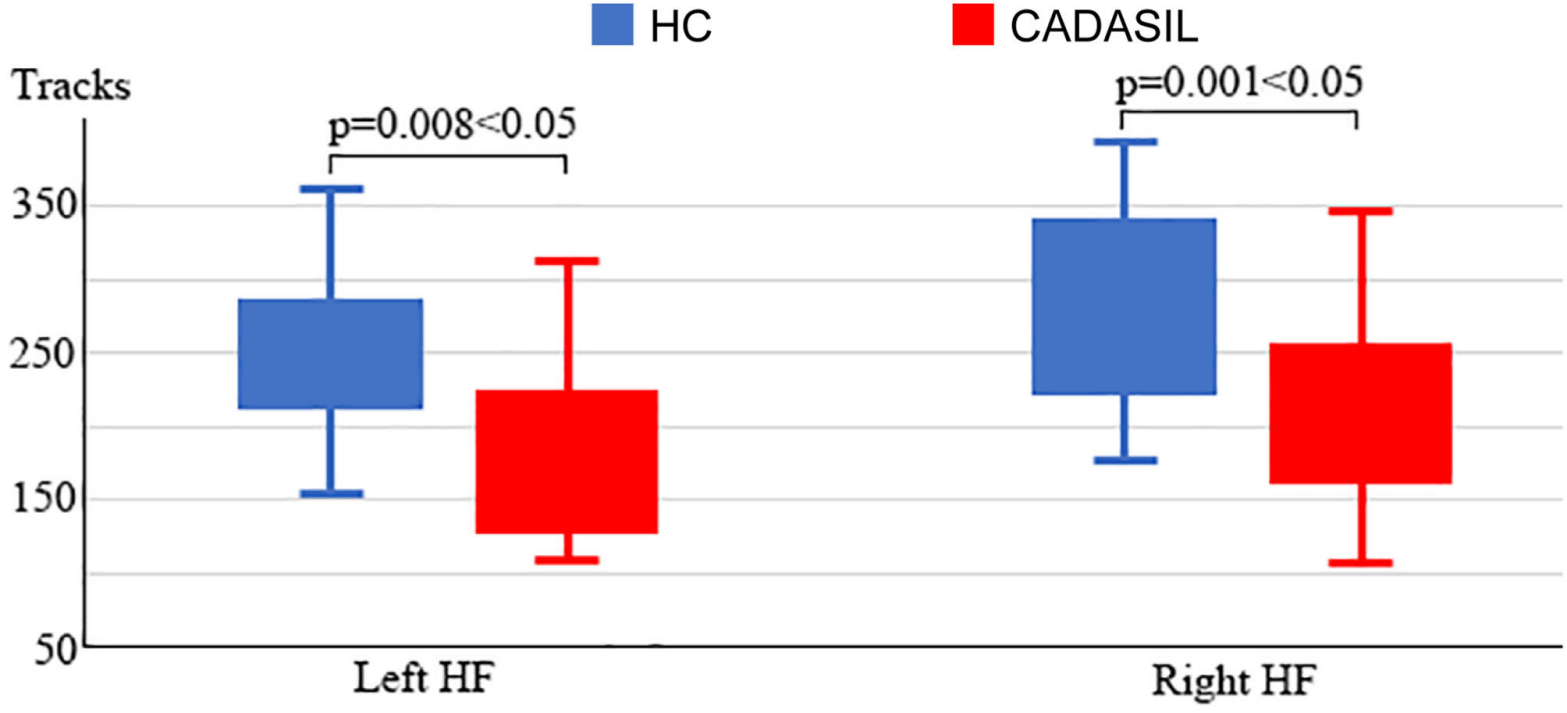
Analysis of fiber tracks in the DMN in CADASIL and control groups. Patients with CADASIL had decreased fiber tracks in the bilateral HF. DMN, default mode network; CADASIL, cerebral autosomal dominant arteriopathy with subcortical infarcts and leukoencephalopathy; HF, hippocampal formation; HC, healthy control.

Compared with the healthy control group, the patients with CADASIL had reduced NT in the bilateral HF (left: 198.86 ± 85.86 vs. 263.33 ± 64.18, respectively, *p* = 0.008; right: 208.05 ± 72.68 vs. 290.24 ± 84.01, respectively, *p* = 0.001) (Figure 4). There was no significant increase in tracks between the CADASIL and healthy control groups.

### Metabolism Analysis of DMN

**Figure 5:**
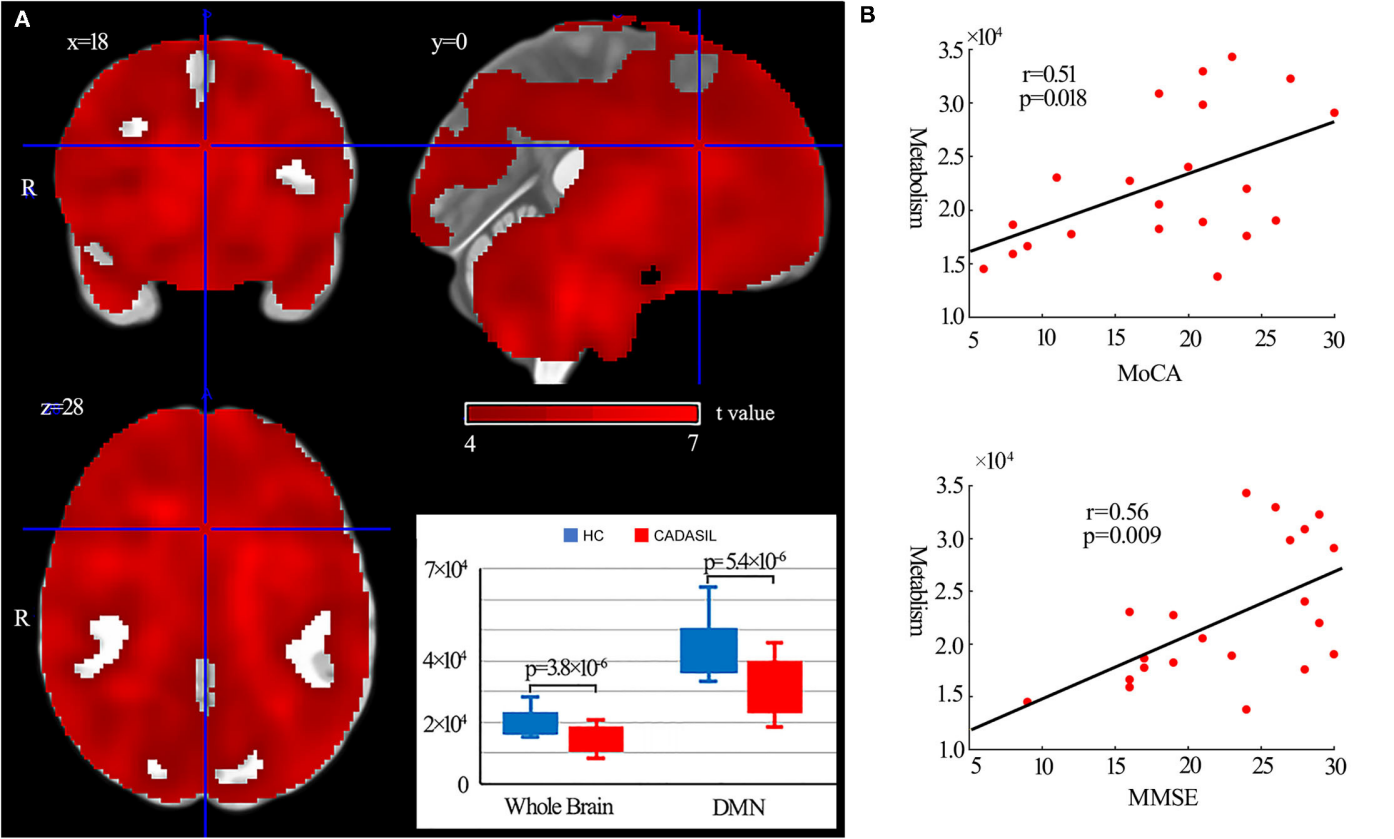
Results of metabolism analysis in the DMN in CADASIL and control groups. **(A)** Metabolism alterations in the CADASIL group. **(B)** Correlations between metabolism across ROIs within the DMN and cognitive scores in the CADASIL group. Patients with CADASIL had decreased metabolism across the whole brain, including the ROIs within the DMN, compared with healthy controls. DMN, default mode network; CADASIL, cerebral autosomal dominant arteriopathy with subcortical infarcts and leukoencephalopathy; ROI, region of interest; HC, healthy control; MoCA, Montreal Cognitive Assessment; MMSE, Mini-Mental State Examination.

Compared with the healthy control group, the patients with CADASIL had decreased glucose metabolism across the ROIs (*p* < 0.00001) (Figure 5A). There was a significant decrease in global metabolism across the whole brain (*p* < 0.00001) (Figure 5A). There was no significant increase in regional metabolism between the two groups.

### Correlation Analysis

In fMRI, the nodal efficiency and degree centrality in the bilateral HF were positively correlated with MoCA and MMSE scores in the patients with CADASIL (*p* < 0.05) (Figure 2B). Similar correlations between the GMV of the bilateral HF and left PHC, and cognitive scores were also detected in the CADASIL group (Figure 3B). There was no significant correlation between the number of tracks and cognitive scores. The levels of metabolism in most ROIs with decreased glucose metabolism were positively correlated with the cognitive scores in the CADASIL group (*p* < 0.01) (Figure 5B and Supplementary Table 3).

## Discussion

The present study was performed to investigate whether the DMN was altered in patients with CADASIL. We integrated the results of multimodal imaging methods, including fMRI, T1-weighted MRI, DTI, and PET, to investigate the changes in functional network properties, GMV, fiber tracks, and glucose metabolism within the DMN in patients with CADASIL. Consistent with our initial hypothesis, the characteristics of DMN represented by these images in the CADASIL group were significantly different compared with those in the healthy control group. Correlation analysis showed that these modifications were associated with cognitive deficits in the CADASIL group. These findings had important implications for the further understanding of CADASIL-related cognitive deficits and could provide potential brain markers for CADASIL.

Previous neuroimaging studies have focused on the whole brain functional or structural changes in CADASIL by a single imaging modality (15, 17, 28). In our study, we focused on the DMN by multi-modal imaging methods. The results in this study corresponded with previous MRI studies on stroke, dementia, migraine, and WMH that showed that functional or structural modifications of DMN are associated with cognitive deficits. In this study, more than 90% of CADASIL subjects had WMH. Although the ratio of migraine, dementia, and migraine was not as high as WMH, these symptoms are also typical of the later clinical manifestations of CADASIL. Notably, in order to fully and thoroughly explore the DMN alterations in CADASIL, except for three MRI modalities, one PET modality which represents the glucose metabolism in the brain was applied to evaluate the network characteristics of DMN. In order to reduce the influence of different subjects on the results, most of the patients with CADASIL (17 of 21) had both PET and MRI modalities.

In the FC analysis, weakened interactions between the HF and MPFC (aMPFC and dMPFC) were detected in both the left and right DMN in patients with CADASIL. Further graph theory analysis of the FC matrixes showed that the changed FC resulted in decreased nodal efficiency and degree centrality of HF and dMPFC in the CADASIL group. These observations indicated that HF and dMPFC within the DMN in the patients with CADASIL have a poor capacity for information propagation, and reduced functional interactions with other regions within the network. Indeed, the HF plays a central node in memory function (29–31) and the dMPFC plays a key role in cognitive performance, including decision making, reward processing, mentalizing, memory, and conceptual processing (6, 31, 32). In addition, functional interactions between the HF and MPFC have been demonstrated to form an important neural circuit for spatial working memory (33–35). Furthermore, functional alterations in the HF or MPFC have been shown to be associated with cognitive deficits in other diseases (36–40). Therefore, our fMRI results demonstrated that the changed FC strength as a particular locus of dysfunction affected the nodal properties of the DMN, which may contribute to cognitive deficits in patients with CADASIL.

To examine structural changes within the DMN in CADASIL, differences between the two groups in GMV and NT of the ROIs were examined. Analyses of both the GMV and NT showed that patients with CADASIL had decreased GMV and NT in the ROI of HF. Further, decreased GMV was found in the left PHC and the bilateral HF in the CADASIL group. Both the PHC and HF are key regions for memory-related cognition (31, 41, 42). In addition, decreased GMV as well as changed FA in the two regions have been reported to be associated with cognitive deficits (15, 43–45). The significant associations between the GMV of HF and cognitive scores were consistent with the results of a previous MRI study in a large cohort (46). The HF belongs to the medial temporal subsystem of the DMN, which, through its interactions with the MPFC, plays a role in a wide range of associative or constructive aspects of mental simulation (6). Indeed, decreased interactions between the MPFC and medial temporal lobe including the hippocampus in resting-state fMRI data, and decreased GMV in the two regions, have been suggested to be associated with cognitive deficits, including working memory, social and emotional processing, and executive function deficits (39, 40, 43–45, 47, 48). The two regions have been suggested to play hub roles in DMN, which is a hub network for advanced cognition (18, 25). Cognitive performance relies on the coordination and collaboration of the activation and deactivation response; if one component fails, the whole system is jeopardized (49, 50). Therefore, the overlap between the results of fMRI and structural MRI indicates that changes of HF within the DMN may play important roles in the cognitive deficits seen in patients with CADASIL, and HF may be a potential brain marker. These MRI results corresponded with a previous DMN study in Fabry disease that showed functional DMN modifications and white matter damage were associated with cognitive deficits (51). However, contrary to the pattern of reduced DMN FC and decreased GMV found in our results, there were no significant differences in GMV, and increased DMN FC was observed in patients with Fabry disease. These differences between CADASIL and Fabry disease implied different pathogenic mechanisms underlying the two conditions, which can be further investigated. In marked contrast to the local variation in MRI results, ^18^F-FDG PET showed that glucose metabolism of each ROI within the whole DMN, and even the whole brain, was decreased in the CADASIL group. Although there have been few PET studies in CADASIL, two independent PET studies reported hypometabolism across the whole brain in the resting state in patients with CADASIL (52, 53), which was consistent with our findings. Furthermore, similar to the results of MRI, metabolism was positively correlated with the cognitive scores in the CADASIL group. These correlations between characteristics of DMN revealed by different imaging modalities and cognitive scores suggested that interior relations may underlie the functional and structural changes within the DMN in patients with CADASIL. However, regional functional or structural disconnection can affect metabolism in other regions and vice versa. Further longitudinal multimodal imaging studies are required to determine the underlying mechanisms.

This study has some limitations, as only resting-state fMRI or PET data were collected, and it is, therefore, unclear whether there were altered patterns of DMN activity or connectivity during task performance in CADASIL, which had been demonstrated in other disorders or with aging (54–57).

## Conclusions

In conclusion, altered functional and structural properties of the DMN were found in patients with CADASIL by multimodal imaging. These cognition-associated changes of HF within the DMN may play important roles in cognitive deficits in CADASIL.

## Data Availability Statement

The raw data supporting the conclusions of this article will be made available by the authors, without undue reservation.

## Ethics Statement

The studies involving human participants were reviewed and approved by Independent Ethics Committee of Shanghai Ninth Hospital, Shanghai Jiao Tong University School of Medicine. The patients/participants provided their written informed consent to participate in this study.

## Author Contributions

JS, FH, and XD: study conception and design. PL, QH, JW, YQ, and YZ: acquisition of data. PL and SB: analysis and interpretation of data. All authors drafting the article and final approval of the version to be published.

## Funding

This research was supported by grants from the National Natural Science Foundation of China (82071282 to JS), the Rare Disease Registration Platform of Shanghai Ninth People's Hospital, Shanghai Jiao Tong University School of Medicine (JYHJB08 to JS), and the Horizontal Research Project from Shanghai Ninth People's Hospital (JYHX2021001 to JS).

## Conflict of Interest

The authors declare that the research was conducted in the absence of any commercial or financial relationships that could be construed as a potential conflict of interest.

## Publisher's Note

All claims expressed in this article are solely those of the authors and do not necessarily represent those of their affiliated organizations, or those of the publisher, the editors and the reviewers. Any product that may be evaluated in this article, or claim that may be made by its manufacturer, is not guaranteed or endorsed by the publisher.
