# Supplementary material for: Altered Default Mode Network Is Associated With Cognitive Impairment in CADASIL as Revealed by Multimodal Neuroimaging
Source: Front Neurol. 2021 Dec 6;12:735033. doi: 10.3389/fneur.2021.735033 (PMC8685443; doi:10.3389/fneur.2021.735033)
Supplement: Supplementary file 1 [file Data_Sheet_1.PDF]

Supplementary materials

Supplementary Table 1. Regions of interest in DMN of CADASIL

	Regions	Abbreviation	Brodmann areas	x	y	z
Left	Dorsal medial prefrontal cortex	dMPFC	9, 32	0	52	26
	Ventral medial prefrontal cortex	vMPFC	11, 24, 25, 32	0	26	-18
	Anterior medial prefrontal cortex	aMPFC	10, 32	-6	52	-2
	Posterior cingulate cortex	PCC	23, 31	-8	-56	26
	Temporal parietal junction	TPJ	40, 39	-54	-54	28
	Lateral temporal cortex	LTC	21, 22	-60	-24	-18
	Temporal pole	TempP	21	-50	14	-40
	Posterior inferior parietal lobule	pIPL	39	-44	-74	32
	Retrosplenial cortex	RSC	29, 30, 19	-14	-52	8
	Parahippocampal cortex	PHC	20, 36, 19	-28	-40	-12
	Hippocampal formation	HF	20, 36	-22	-20	-26
Right	Dorsal medial prefrontal cortex	dMPFC	9, 32	0	52	26
	Ventral medial prefrontal cortex	vMPFC	11, 24, 25, 32	0	26	-18
	Anterior medial prefrontal cortex	aMPFC	10, 32	6	52	-2
	Posterior cingulate cortex	PCC	23, 31	8	-56	26
	Temporal parietal junction	TPJ	40, 39	54	-54	28
	Lateral temporal cortex	LTC	21, 22	60	-24	-18
	Temporal pole	TempP	21	50	14	-40
	Posterior inferior parietal lobule	pIPL	39	44	-74	32
	Retrosplenial cortex	RSC	29, 30, 19	14	-52	8
	Parahippocampal cortex	PHC	20, 36, 19	28	-40	-12
	Hippocampal formation	HF	20, 36	22	-20	-26

Note: Coordinates were based on the Montreal Neurological Institute coordinate system. Brodmann areas listed approximate locations for reference. DMN, default mode network; CADASIL, cerebral autosomal dominant arteriopathy with subcortical infarcts and leukoencephalopathy.

Supplementary Table 2. Mathematical definitions of complex network measures

Measures	Definitions
Basic concepts and measures: *N* is the set of all nodes in the network, and *n* is the number of nodes. *L* is the set of all links in the network, and *l* is number of links. (i, j) represent the link between nodes i and j, $(i, j \in N)$. w_{ij}, the Pearson correlation coefficient between regions i and j, represents the connection weights between nodes i and j. a_{ij}represents the connection status between i and j for weighted network, $a_{ij} = correlation(i,j)$.	
Nodal degree: a measurement of connectivity of a node with the rest of nodes in a network	Degree of i, $k_i = \sum_{j\in N} a_{ij}$
Nodal degree centrality	Degree centrality of i, $C_D(i) = \frac{\sum_{j=1} a_{ij}(i\neq j)}{n-1}$
Shortest path length: a basis for measuring integration	Shortest weighted path length between i and j, $d_{ij} = \sum_{a_{uv}\in gi\leftrightarrow j} f(w_{uv})$, where f is a map from weight to length and $gi \leftrightarrow j$ is the set of shortest path between i and j.
Nodal clustering coefficient: a basis for measuring segregation, reflects the local interconnectivity or cliques among the neighbors of a given node	Clustering coefficient of i, $C_i = \sum_{i\in N} \frac{2t_i}{k_i(k_i-1)}$ $t_i = \frac{1}{2}\sum_{j,h\in N} (w_{ij}w_{ih}w_{jh})^{1/3}$
Clustering coefficient of the network	$C = \frac{1}{n}\sum_{i\in N} C_i$
Characteristic path length	$L = \frac{1}{n}\sum_{i\in N} \frac{\sum_{j\in N,j\neq i} d_{ij}}{n-1}$
Nodal efficiency: the ability of information propagation between a given node i with the rest of nodes in a network	Efficiency of i, $E_i = \frac{\sum_{j\in N,j\neq i}(d_{ij})^{-1}}{n-1}$
Global efficiency: the ability of information transmission of a network at the global level	$E = \frac{1}{n}\sum_{i\in N} E_i$
Nodal local efficiency: the ability of information propagation between a given node i with its near nodes in a network	Local efficiency of i, $E_{loc,i} = \frac{\sum_{j,h\in N,j\neq i}(w_{ij}w_{ih}[d_{jh}(N_i)]^{-1})^{1/3}}{k_i(k_i-1)}$
Global local efficiency: the ability of information transmission of a network at the local level	$E_{loc} = \frac{1}{2}\sum_{i\in N} E_{loc,i}$
Betweenness centrality: the influence of the node over information flow between all the other nodes in the network	$b_i = \frac{1}{(n-1)(n-2)}\sum_{\substack{h,j\in N \\ h\neq j\neq i}} \frac{\rho_{hj}(i)}{\rho_{hj}}$, where ρ_{hj} is the number of shortest paths between h and j, and $\rho_{hj}(i)$ is the number of shortest paths between h and j that pass through i

Small-worldness: in both cases, small-world networks often have $S \geq 1$	$S = \frac{C/C_{rand}}{L/L_{rand}}$
Assortativity coefficient: a measure of the correlation between the degree of a node and the mean degree of its nearest neighbors	$r = \frac{l^{-1}\sum_{(i,j)\in L} w_{ij}k_ik_j - \left[l^{-1}\sum_{(i,j)\in L}\frac{1}{2}w_{ij}(k_i + k_j)\right]^2}{l^{-1}\sum_{(i,j)\in L}\frac{1}{2}w_{ij}(k_i^2 + k_j^2) - \left[l^{-1}\sum_{(i,j)\in L}\frac{1}{2}w_{ij}(k_i + k_j)\right]^2}$
Hierarchy: A large positive value of β means a hierarchical network where nodes with high degree are connected predominantly to nodes not otherwise connected to each other and vice versa	$C = k^{-\beta}$
Synchronization: the property of a network to synchronize in dynamics among coupled oscillators	$Sy = \frac{\lambda_2}{\lambda_N}$, where λ_2 and λ_N are the second smallest and the largest eigenvalue of the coupling matrix G which is defined as $G_{ij} = \delta_{ij}\sum_{j=1}^{N} w_{ij} - w_{ij}$

Supplementary Table 3. Correlations between metabolism of each ROI and cognitive scores in CADASIL

Left		aMPFC	PCC	dMPFC	TPJ	LTC	TempP	vMPFC	pIPL	RSC	PHC	HF
MoCA	left	0.48/0.03	0.53/0.01	0.53/0.01	0.55/0.01	0.42/0.06	0.44/0.05	0.45/0.04	0.60/0.004	0.48/0.03	0.42/0.06	0.52/0.02
	right	0.55/0.009	0.53/0.01	0.53/0.01	0.51/0.02	0.39/0.08	0.40/0.07	0.45/0.04	0.52/0.02	0.40/0.07	0.41/0.07	0.44/0.05
MMSE	left	0.54/0.01	0.56/0.008	0.57/0.005	0.57/0.007	0.46/0.04	0.44/0.05	0.52/ 0.02	0.64/0.002	0.47/0.03	0.44/0.05	0.58/0.005
	right	0.60/0.004	0.57/0.007	0.59/0.005	0.61/0.003	0.52/0.02	0.46/0.04	0.52/ 0.02	0.58/0.005	0.41/0.06	0.46/0.03	0.50/0.02

Note: Data before backslash represented correlation coefficients and data after backslash represented the corresponding *p* values after FDR corrected. ROI, region of interest; CADASIL, cerebral autosomal dominant arteriopathy with subcortical infarcts and leukoencephalopathy; FDR, false discovery rate; MoCA, Montreal Cognitive Assessment; MMSE, Mini-Mental State Examination; aMPFC, anterior medial prefrontal cortex; PCC, posterior cingulate cortex; dMPFC, dorsal medial prefrontal cortex; TPJ, temporal parietal junction; LTC, lateral temporal cortex; TempP, temporal pole; vMPFC, ventral medial prefrontal cortex; pIPL, posterior inferior parietal lobule; RSC, retrosplenial cortex; PHC, parahippocampal cortex; HF, hippocampal formation.

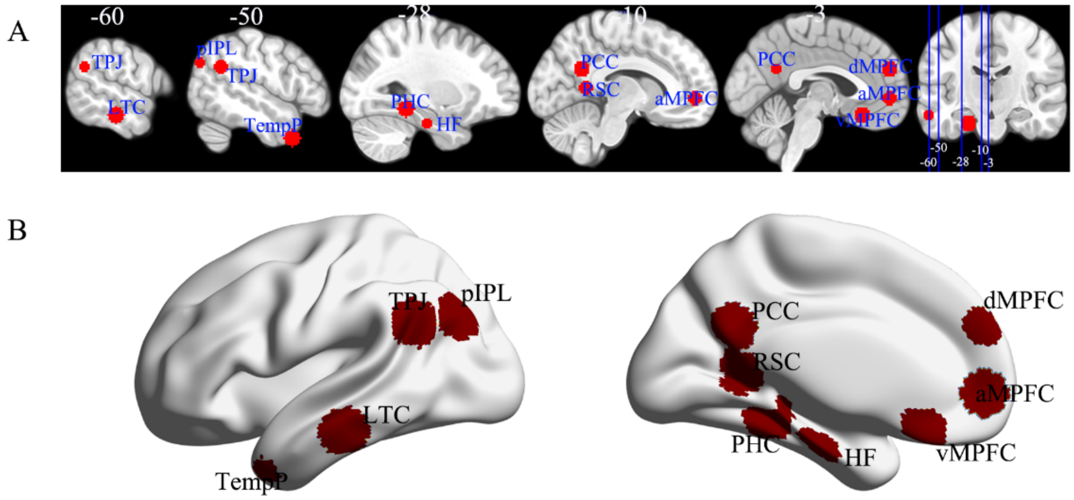

Supplementary Figure 1. Regions of interest in DMN of CADASIL. (A) Regions were shown overlapping on a structural MRI template. (B) Regions were also projected onto a surface template. DMN, default mode network; CADASIL, cerebral autosomal dominant arteriopathy with subcortical infarcts and leukoencephalopathy; MRI, magnetic resonance imaging; dMPFC, dorsal medial prefrontal cortex; vMPFC, ventral medial prefrontal cortex; aMPFC, anterior medial prefrontal cortex; PCC, posterior cingulate cortex; TPJ, temporal parietal junction; LTC, lateral temporal cortex; TempP, temporal pole; pIPL, posterior inferior parietal lobule; RSC, retrosplenial cortex; PHC, parahippocampal cortex; HF, hippocampal formation.

Supplementary Figure 2. P values of comparisons between CADASIL and controls in FC.

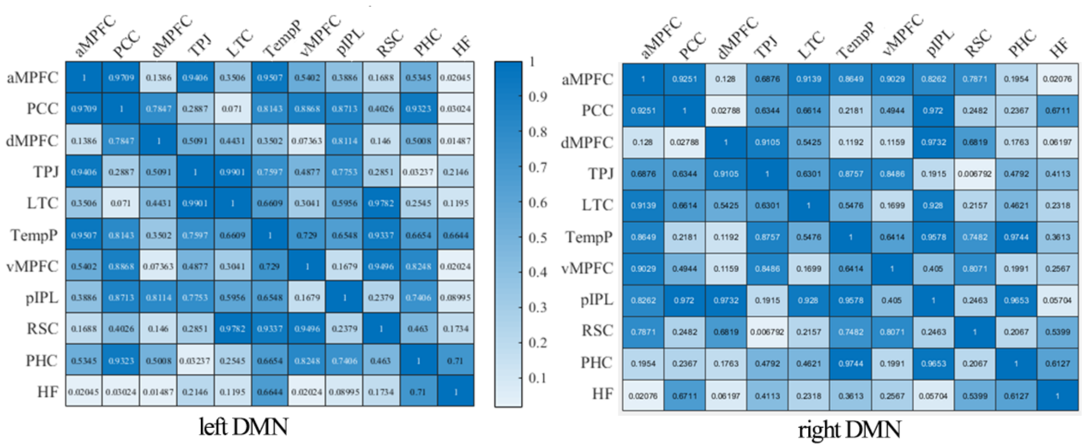

Color bar represented *p* values. CADASIL, cerebral autosomal dominant arteriopathy with subcortical infarcts and leukoencephalopathy; FC, functional connectivity; aMPFC, anterior medial prefrontal cortex; PCC, posterior cingulate cortex; dMPFC, dorsal medial prefrontal cortex; TPJ, temporal parietal jucnction; LTC, lateral temporal cortex; TempP, temporal pole; vMPFC, ventral medial prefrontal cortex; pIPL, posterior inferior parietal lobule; RSC, restrosplenial cortex; PHC, parahippocampal cortex; HF, hippocampal formation.
